# Mixed Pain: Toward a Consensus Definition and a Mechanism‐Based Framework

**DOI:** 10.1002/ejp.70356

**Published:** 2026-08-06

**Authors:** Rainer Freynhagen, Bart Morlion, Antonio Alcántara Montero, Daniel Ciampi de Andrade, Andrés Hernández‐Ortiz, Paul W. Hodges, Sasikaan Nimmaanrat, Esther M. Pogatzki‐Zahn, Milton Raff, Roberto D. Rey, Solomon Tesfaye, Andrea Truini, Giustino Varrassi, Ralf Baron

**Affiliations:** ^1^ Department of Anaesthesiology, Critical Care Medicine & Pain Therapy Benedictus Hospital Feldafing & Tutzing Feldafing Germany; ^2^ Department of Anaesthesiology Klinikum Rechts der Isar, Technische Universität München Munich Germany; ^3^ The Leuven Centre for Algology and Pain Management University Hospitals Leuven Leuven Belgium; ^4^ Department of Cardiovascular Sciences, Section Anaesthesiology and Algology KU Leuven‐University of Leuven Leuven Belgium; ^5^ Servicio Extremeño de Salud Trujillo Spain; ^6^ Center for Neuroplasticity and Pain (CNAP), Department of Health Science and Technology, Faculty of Medicine Aalborg University Aalborg Denmark; ^7^ Pain and Palliative Medicine Instituto Nacional de Ciencias Médicines y Nutrición Salvador Zubirán Mexico City Mexico; ^8^ Centre for Innovation in Pain and Health Research (CIPHeR) The University of Queensland Brisbane Australia; ^9^ Department of Anesthesiology Prince of Songkla University Hat Yai Songkhla Thailand; ^10^ Department of Anaesthesiology, Intensive Care, and Pain Medicine Univerisity Hospital Münster Germany; ^11^ Pain Clinic, Christiaan Barnard Memorial Hospital Cape Town South Africa; ^12^ Instituto Argentino de Investigación Neurológica Universidad de Buenos Aires Buenos Aires Argentina; ^13^ Diabetes Research Unit Sheffield Teaching Hospitals NHS Foundation Trust Sheffield UK; ^14^ Department of Human Neuroscience Sapienza University Rome Italy; ^15^ Fondazione Paolo Procacci Rome Italy; ^16^ Division of Neurological Pain Research and Therapy, Department of Neurology Christian‐Albrechts‐Universität Kiel Kiel Germany

## Abstract

**Background:**

For decades, clinicians and researchers have struggled to classify pain conditions that do not fit neatly into the nociceptive, neuropathic or nociplastic frameworks. Many common disorders—from chronic low back pain to cancer pain and osteoarthritis—exhibit overlapping mechanisms and have long fallen into the ‘grey zone’ of mixed pain. This area has intrigued researchers yet frustrated clinicians due to the absence of a clear, unified definition.

**Methods:**

Through an international consensus process led by global leading experts, mixed pain has now been defined as pain that is associated with a lesion, disease or disorder resulting in an overlap of at least two mechanistic pain descriptors (nociceptive, neuropathic or nociplastic).

**Conclusions:**

This new definition may help provide the context needed to align research, refine diagnosis and design mechanism‐based therapies for patients with mixed pain. It marks an important step towards conceptual clarification—from confusion to consensus, from overlap to action—transforming mixed pain from an ambiguous concept into a potential framework of modern pain taxonomy.

**Significance Statement:**

This work provides an internationally unified consensus definition of mixed pain, addressing inconsistency in pain terminology and mechanistic classification. By defining mixed pain as the overlap of at least two mechanistic pain descriptors (nociceptive, neuropathic or nociplastic), the framework clarifies conceptual ambiguity while stressing the importance of identifying the underlying pain descriptors, supports a mechanism‐based assessment and may facilitate more individualized treatment approaches in complex pain conditions while providing a practical foundation for future research, validation and clinical application.

## 1 Introduction

Despite an increase in attention on the topic of ‘mixed pain’ (Leoni et al. 2025; Saracoglu et al. 2025; Varrassi et al. 2025), it remains a concept without a formal, accepted definition. This conceptual ambiguity translates into clinical uncertainty, as many patients with conditions such as low back pain, osteoarthritis, or cancer pain do not experience just one type of pain, as characterized by the mechanistic pain descriptors nociceptive, neuropathic, or nociplastic pain. Instead, they experience an overlap of these mechanistic pain descriptors, falling into the undefined categorization of having ‘mixed pain’ (Freynhagen et al. 2019).

The absence of standardized terminology and defined criteria make it difficult to identify and appropriately manage patients living with these conditions, posing a clear challenge to clinical practice (Freynhagen et al. 2019, 2020; Leoni et al. 2025).

The understanding that these different mechanistic pain descriptors can exist in combination has to be clearly communicated to the pain community, so an easy‐to‐use definition is a prerequisite. Additionally, a lack of acceptance by larger regulatory bodies and scientific societies (IASP 2011; Freynhagen et al. 2019; Treede et al. 2019; Fernandez‐Fairen et al. 2023) contributes to the hurdles of harmonizing research on conditions with mixed pain, resulting in a lack of established clinical data in these patient populations. With an urgent unmet need to define mixed pain and a rapidly growing interest in this topic (Leoni et al. 2025; Saracoglu et al. 2025; Varrassi et al. 2025), the question still remains: ‘What is mixed pain?’

The term ‘mixed pain’ has been used in the literature for many years, often in reference to conditions in which nociceptive and neuropathic pain contribute simultaneously to the pain experience (Grond et al. 1999; Freynhagen and Baron 2009; Förster et al. 2013; Clauw and Hassett 2017; Ponce et al. 2019). However, its meaning has remained variable and sometimes ambiguous, reflecting the absence of a shared conceptual framework (Portenoy et al. 2006; Roosink et al. 2010; Freynhagen et al. 2019; Leoni et al. 2025). Despite increasing clinical recognition that multiple mechanistic pain descriptors frequently coexist within individual patients, terminology describing such situations remains inconsistent.

Two formal attempts have been made to define mixed pain (Table 1). The first, by Freynhagen et al. (2019) aimed to provide a foundation toward a more widely accepted mechanistic and/or clinical definition. The subsequent Latin American Consensus sought to develop a simplified, culturally adapted version of this definition (Fernandez‐Fairen et al. 2023). However, by condensing and thereby oversimplifying the definition, a conceptual inaccuracy was introduced, with the implication that all three pain phenotypes are required to constitute mixed pain. The existence of two definitions—one preliminary and foundational, the other concise but conceptually flawed—has made it evident that a unified description of mixed pain is urgently needed. The aim of the present perspective is therefore not to introduce a new concept, but rather to clarify and harmonize the use of an already widely employed term through an international consensus process.

**TABLE 1:** Published definitions of mixed pain.

Group	Year	Definition
First international consensus attempt (Freynhagen et al. 2019)	2019	‘Mixed pain is a complex overlap of the different known pain types (nociceptive, neuropathic, nociplastic) in any combination, acting simultaneously and/or concurrently to cause pain in the same body area. Either mechanism may be more clinically predominant at any point of time. Mixed pain can be acute or chronic’
Latin American consensus committee (Fernandez‐Fairen et al. 2023)	2023	‘Mixed pain is a complex condition including components of nociceptive, neuropathic and nociplastic pain’

## 2 Methods

### 2.1 Study Overview

This study employed a structured consensus process based on Delphi methodology to establish an internationally harmonized, clinically relevant, and scientifically robust definition of mixed pain. This approach was selected for its recognized capacity to systematically achieve expert consensus on complex and evolving concepts lacking universally accepted definitions (Hsu and Sandford 2007; Keeney et al. 2011; Junger et al. 2017). The methodology followed established recommendations for Delphi studies and consensus development, incorporating transparent procedures, anonymous voting, iterative feedback and predefined consensus thresholds (Junger et al. 2017). This initiative represents the culmination of years of multidisciplinary collaboration, scientific debate, advisory board activities and critical appraisal of the literature addressing the conceptual, pathophysiological, and clinical dimensions of mixed pain (Freynhagen et al. 2019; Varrassi et al. 2025).

### 2.2 Expert Panel Selection

An international multidisciplinary panel of 14 recognized experts in pain medicine and related fields was assembled. Selection criteria included internationally acknowledged expertise in pain management, substantial scientific contributions, active participation in multidisciplinary care, and prior involvement in mixed pain research. The resulting panel encompassed specialists from pain medicine, anesthesiology, neurology, rheumatology, endocrinology, rehabilitation medicine, primary care and translational pain sciences, ensuring broad scientific representation and global applicability of the final definition. Although five experts could not participate in the synchronous meeting and voting process, they contributed to the preparatory and validation phases and participated in the second vote to reach the final consensus statement.

### 2.3 Preparatory Phase

Prior to the live discussion, participants received a standardized scientific dossier comprising key publications, previous consensus statements, and summaries of earlier discussions on mixed pain, including definitions, terminology, pathophysiological frameworks, diagnostic considerations and clinical applications. Participants also reviewed current International Association for the Study of Pain (IASP) definitions to ensure consistency with internationally accepted terminology regarding nociceptive, neuropathic, and nociplastic mechanistic pain descriptors (IASP 2011).

### 2.4 Structured Consensus Design

The structured consensus process comprised iterative rounds of structured discussions followed by anonymous voting. Participants first examined the conceptual boundaries of mixed pain; its relationship with nociceptive, neuropathic and nociplastic pain; and its implications for clinical practice and future research. Subsequently, existing definitions were systematically evaluated (Table 1), focusing on the scientific validity, clarity, and appropriateness of terminology related to mechanistic pain descriptors, temporal relationships, anatomical considerations, underlying biological processes/pain mechanisms and pain duration. A preliminary definition was then collaboratively developed and submitted for formal evaluation.

### 2.5 Consensus and Voting Procedures

Consensus was established through anonymous electronic voting using an Audience Response System, minimizing the influence of dominant opinions and social desirability bias, in accordance with established Delphi methodology (Hsu and Sandford 2007; Keeney et al. 2011; Junger et al. 2017). Predefined thresholds were established a priori: Components receiving ≥ 80% affirmative votes were accepted, those receiving 60%–79% underwent further discussion and re‐voting, and those receiving < 60% were excluded. Following consensus on individual components, the definition underwent editorial refinement to optimize scientific accuracy, linguistic clarity, and international applicability, and was subsequently submitted to a second round of anonymous voting using the same criteria.

Post‐meeting, additional discussions on the definition transpired between authors, and a final anonymous virtual vote occurred on a revised definition through Survey Monkey, in which all 14 authors participated, utilizing the same voting thresholds stated above.

### 2.6 Outcome Measures

The primary outcome was the development of a consensus‐based definition of mixed pain characterized by scientific accuracy, conceptual clarity, international applicability, and clinical usability.

### 2.7 Methodological Rigour

Several methodological safeguards were implemented to enhance the validity and reliability of the consensus process, including international multidisciplinary representation, standardized preparatory materials, iterative discussion and refinement, anonymous voting, and predefined consensus thresholds, all recognized as essential components of high‐quality Delphi studies (Hsu and Sandford 2007; Keeney et al. 2011; Junger et al. 2017). This structured consensus process was designed to generate a concise, evidence‐informed and universally applicable definition of mixed pain, fostering future research and international harmonization of this emerging pain construct.

## 3 Results

### 3.1 Core Components, Structure and Preliminary Definition

A central element of the revised mixed pain definition was the requirement that *at least* 2 of the 3 mechanistic pain descriptors—nociceptive, neuropathic or nociplastic—be present, thereby clarifying all possible permutations of these descriptors. ‘Overlap,’ defined as ‘to occupy the same area in part’ (Merriam‐Webster n.d.) or ‘to coincide partly’ (Oxford English Dictionary n.d.), was deemed sufficient for conveying that the different mechanistic pain descriptors comprising mixed pain may occur at the same instant (simultaneously) or at the same time with an overlap in duration (concurrently). It also infers that a single mechanistic pain descriptor may be more clinically predominant at any point of time.

Though the previous definition by Freynhagen et al. described the need for pain to be localized in the same body area (Table 1; Freynhagen et al. 2019), this is not in fact inclusive of all types of mixed pain, for example, back pain in which pain radiates down the leg and/or up into the back. However, the terminology ‘pain arises from a lesion, disease, or disorder/syndrome’ is a more accurate description. Additionally, the use of the indefinite article ‘a’ implies that the pain arises from one pathological entity or specific disease, thus excluding co‐existing pain.

Rather than introducing additional complexity by including duration‐based distinctions, ‘acute’ and ‘chronic’ were excluded from the revised definition, keeping the focus of the definition on the overlap of mechanistic pain descriptors.

These key individual components, as well as the full definition, were advanced to a formal vote (Table 2). A strong majority agreement for inclusion (≥ 80% alignment) was achieved on the individual components that were then assembled into the full definition, and consensus (100% alignment) was obtained on that initial mixed pain definition: ‘Mixed pain is pain that arises from a lesion, disease or disorder resulting in an overlap of at least two pain mechanisms (nociceptive, neuropathic or nociplastic).’

**TABLE 2:** Anonymous voting results on individual components and final definition of mixed pain.

Individual component	Voting results (*n* = 10)
Pain that arises from	Yes: 100% No: 0%
A lesion	Yes: 100% No: 0%
A disease	Yes: 100% No: 0%
A disorder	Yes: 90% No: 10%
A syndrome	Yes: 10% No: 90%
A syndrome/disorder	Yes: 0% No: 100%
Resulting in an overlap of at least two pain mechanisms (nociceptive, neuropathic, or nociplastic)	Yes: 100% No: 0%

Full definition vote on‐site	(*n* = 10)
Mixed pain is pain that arises from a lesion, disease, or disorder resulting in an overlap of at least two pain mechanisms (nociceptive, neuropathic, or nociplastic)	Yes: 100% No: 0%

Final virtual definition vote	(*n* = 14)
Mixed pain is pain that is associated with a lesion, disease, or disorder resulting in an overlap of at least two mechanistic pain descriptors (nociceptive, neuropathic, or nociplastic)	Yes: 100% No: 0%

*Note:* Decision rules: Strong majority agreement included in definition (‘Yes’: ≥ 80%); no consensus reassessed and revoted (‘Yes’: 60%–79%); dismissed from definition (‘Yes’: < 60%).

### 3.2 Refinement of the Definition

Further discussion and refinement of the definition was undertaken with the replacement of ‘arises from’ with ‘associated with’. In the case that nociplastic pain emerges or overlaps with a nociceptive or neuropathic condition (e.g., painful radiculopathy that later develops regional nociplastic pain), this avoids the implication that the pain results solely from the ‘lesion’. A second update recommended replacing ‘pain mechanisms’ with ‘mechanistic pain descriptors’, as the terms nociceptive, neuropathic, and nociplastic are intended as descriptors rather than being mechanisms themselves. This is particularly important for nociplastic pain, as there is no single ‘nociplastic mechanism’, but a range of underlying processes that are presumed to contribute to it.

**FIGURE 1:**
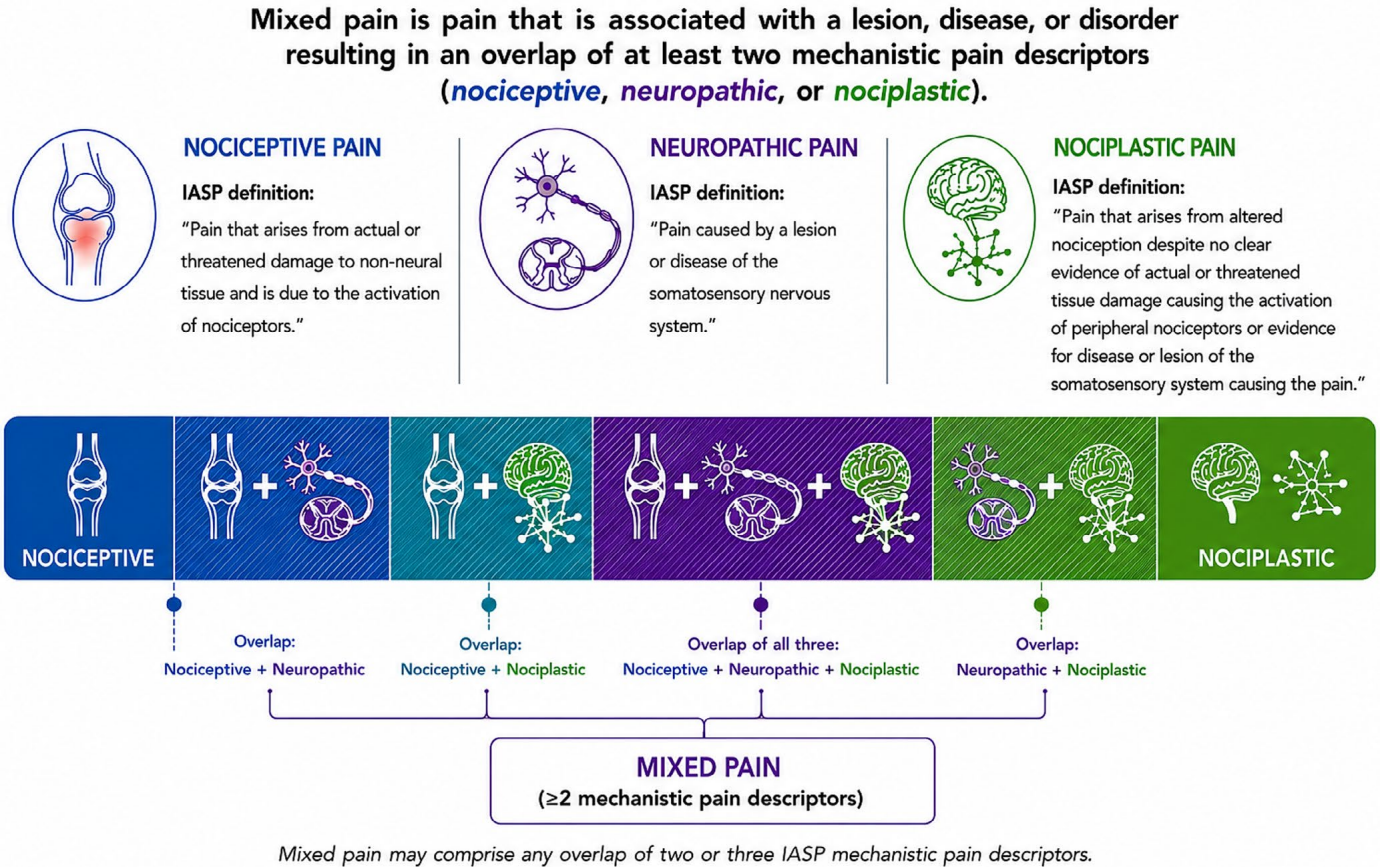
Proposed consensus definition of mixed pain.

The following definition was proposed and reached full consensus after voting by all authors:

Mixed pain is pain that is associated with a lesion, disease, or disorder resulting in an overlap of at least two mechanistic pain descriptors (nociceptive, neuropathic, or nociplastic). (Figure 1)

## 4 Discussion

Mixed pain constitutes a distinct, clinical phenotype that may have therapeutic implications and potential clinical relevance. Many prevalent pain conditions, including chronic low back pain, osteoarthritis, cancer pain, CRPS, post‐surgical pain and even conditions such as fibromyalgia, cannot be adequately characterized by a single mechanistic pain descriptor alone (Freynhagen and Baron 2009; Clauw and Hassett 2017; Mulvey et al. 2017; Pogatzki‐Zahn et al. 2017; Freynhagen et al. 2019; Saracoglu et al. 2025). While the individual descriptors remain essential for mechanistic understanding, there is currently no broadly accepted terminology that formally captures their overlap. The mixed pain framework described above provides a clinically meaningful framework with applications across patient settings and may facilitate improved communications between clinicians and researchers. Identification of patients experiencing mixed pain, and thus more than one mechanistic pain descriptor, allows for better patient phenotyping and stratification in clinical trials, ensuring appropriate patient enrollment and analysis in trials with therapies targeting only one mechanistic pain descriptor. Additionally, the recognition of these patients in clinical practice may aid in alerting clinicians to the biological complexity of their patients' pain, resulting in better‐informed treatment strategies that are targeted to each mechanistic pain descriptor.

However, it is important to note that mixed pain does not represent a separate diagnostic category or a fourth mechanistic pain descriptor, but instead, it acknowledges the coexistence and potential interaction of more than one underlying mechanistic pain descriptor (nociceptive, neuropathic or nociplastic) contributing to the patient's pain experience within a clinical syndrome. For example, a patient with osteoarthritis may exhibit predominantly nociceptive pain, whereas another patient with the same disease may also fulfil criteria for neuropathic pain or show features consistent with nociplastic pain. In such cases, the term mixed pain may be used to indicate overlap of these mechanistic pain descriptors within the clinical disease, while the underlying diagnosis remains unchanged. Regardless, identification of the contributing mechanistic pain descriptors remains essential and should accompany the use of the term whenever possible, and as such, mixed pain is not intended to serve as a label for unknown pain mechanisms.

The intention of the present proposal is not to replace existing mechanistic descriptors or reduce the precision of mechanistic pain classification, but to provide a framework that acknowledges that multiple pain mechanisms frequently coexist within the same clinical condition. It is crucial to recognize that these mechanisms may not only occur simultaneously, but may also influence each other, shaping symptom expression, disease course and treatment response. Clinical studies in chronic low back pain and osteoarthritis have demonstrated that the presence of neuropathic pain is negatively associated with the effectiveness of treatments for nociceptive pain (Romanò et al. 2009; Soni et al. 2016, 2019; Magni et al. 2021), and as such, these patients with mixed pain should be distinguished from those with purely nociceptive pain. Recent research using electrodermal activity measurements in patients with cancer pain demonstrated that specific physiological parameters were associated with both pain intensity and distinct pain phenotypes, particularly mixed pain, suggesting objectively measurable differences compared with nociceptive or neuropathic pain alone (Cascella et al. 2026). While these findings require further validation, they provide initial objective evidence that mixed pain may represent more than a simple coexistence of mechanisms and reflect a distinct pattern of mechanistic interaction. Recognizing mixed pain may therefore enhance, rather than reduce, clinical reasoning by explicitly highlighting situations in which multiple mechanisms contribute to the pain experience and may require integrated therapeutic strategies (Varrassi et al. 2025).

The absence of a universally accepted definition of mixed pain has hindered research and clinical practice, and has created inconsistencies in diagnosis, communication, and treatment (Freynhagen et al. 2019, 2020; Leoni et al. 2025).

## 5 Limitations

The design of this consensus may be subject to limitations. A formal Delphi process that included multiple independent survey rounds prior to the live meeting and first vote was not performed. Additionally, all contributing members were involved in direct discussions with one another, both live and virtually, potentially introducing bias into the discussion; however, an anonymous voting process was maintained throughout the structured consensus process.

## 6 Conclusions

The consensus achieved by these international pain experts represents an important contribution: a clear, potentially broadly applicable definition of mixed pain. This new definition provides a framework for further discussion and research, and it signals an important step forward in the global understanding of more complex pain mechanisms. Its ultimate value, however, will depend on broad validation across settings—including clinical validation that identification of this phenotype leads to better clinical outcomes—and on formal endorsement and inclusion in the official terminology of leading pain societies (IASP 2011; Treede et al. 2019), ensuring worldwide recognition, consistency and sustainable progress in patient‐centered pain management.

## Author Contributions

The live consensus meeting with initial vote was attended by R.F., B.M., A.H.‐O., S.N., M.R., R.R., S.T., A.T., G.V., and R.B. All authors critically reviewed the final definition and corresponding reasoning for the updated language and participated in the virtual vote. R.F. had a primary role in preparing the manuscript, which was edited by all authors. All authors have approved the final version of the manuscript and agree to be accountable for all aspects of the work.

## Funding

This work was supported by Viatris Inc.

## Disclosure

*Use of artificial intelligence*: No.

*Plagiarism declaration*: We hereby declare that this paper is our own work, except where acknowledged, and has not been submitted elsewhere.

## Conflicts of Interest

R. Baron consults with Pfizer, Sanofi Aventis, Grünenthal, Lilly, Novartis, Bristol‐Myers Squibb, Biogenidec, AstraZeneca, Daiichi Sankyo, Glenmark, Seqirus, Teva, Genentech, Mundipharma, Galapagos NV, Kyowa Kirin, Vertex, Biotest, Celgene, Desitin, Regeneron, Theranexus, Abbott, Bayer, Akcea, Asahi Kasei, AbbVie, Air Liquide Sante, Alnylam, Lateral, Hexal, Angelini, Janssen, SIMR, Confo, Merz, Neumentum, F Hoffmann‐La Roche, AlgoTherapeutix, Nanobiotix, AmacaThera, Hoba, Heat2Move, Resano, Esteve, Aristo, Viatris, and Vertanical. B. Morlion received honoraria for speaking and consultancy from Krka, Viatris, Haleon, Opella, Novartis, Heat2Move, Alkaloid, and Grünenthal. R. Freynhagen received consulting, advisory board, speaking fees from Aristo, Augustin, Grünenthal, P&G, Tris, and Viatris. E.M. Pogatzki‐Zahn received financial support for research activities from Grünenthal; advisory and lecture fees from Grünenthal, MSD/MERCK, and Medtronic; and scientific support from the DFG, BMFTR, G‐BA, and Innovative Medicines Initiative 2 Joint Undertaking under grant agreement No 777500. The Joint Undertaking receives support from EU Horizon 2020 research and innovation program and EFPIA. E.M. Pogatzki‐Zahn was not in Bangkok at the meeting and did not receive any support from Viatris for any contributions to this manuscript. S. Tesfaye consulted for Bayer, NeuroPN, Worwag, Angelini, Grünenthal, Nevro, Viatris, Merz, AstraZeneca, and Confo, and received honoraria from Worwag, Novo Nordisk, Merk, AstraZeneca, Nevro, P&G, Viatris, Berlin‐Chemie, Grünenthal, and Medtronic. A. Truini received honoraria for speaking and consultancy, research funding from Grünenthal, Viatris, Angelini, Epitech, Amgen, AbbVie, Exeltis, and Lusofarmaco. A. Alcántara Montero, D. Ciampi de Andrade, A. Hernández‐Ortiz, P.W. Hodges, S. Nimmaanrat, M. Raff, R.D. Rey, and G. Varrassi have no conflicts of interest to declare.
